# Supplementary material for: Effect of exercise intervention on health-related quality of life in middle-aged and older people with osteoporosis: a systematic review and meta-analysis
Source: PeerJ. 2025 Jan 31;13:e18889. doi: 10.7717/peerj.18889 (PMC11789655; doi:10.7717/peerj.18889)
Supplement: Supplemental Information 2 [file peerj-13-18889-s002.docx]

((((("Osteoporosis"[Mesh]) OR (((((((((((((((((((((Osteoporoses[Title/Abstract]) OR (Osteoporosis, Post-Traumatic[Title/Abstract])) OR (Osteoporosis, Post Traumatic[Title/Abstract])) OR (Post-Traumatic Osteoporoses[Title/Abstract])) OR (Post-Traumatic Osteoporosis[Title/Abstract])) OR (Osteoporosis, Senile[Title/Abstract])) OR (Osteoporoses, Senile[Title/Abstract])) OR (Senile Osteoporoses[Title/Abstract])) OR (Osteoporosis, Involutional[Title/Abstract])) OR (Senile Osteoporosis[Title/Abstract])) OR (Osteoporosis, Age-Related[Title/Abstract])) OR (Osteoporosis, Age Related[Title/Abstract])) OR (Bone Loss, Age-Related[Title/Abstract])) OR (Age-Related Bone Loss[Title/Abstract])) OR (Age-Related Bone Losses[Title/Abstract])) OR (Bone Loss, Age Related[Title/Abstract])) OR (Bone Losses, Age-Related[Title/Abstract])) OR (Age-Related Osteoporosis[Title/Abstract])) OR (Age Related Osteoporosis[Title/Abstract])) OR (Age-Related Osteoporoses[Title/Abstract])) OR (Osteoporoses, Age-Related[Title/Abstract]))) AND ((("Aged"[Mesh]) OR (Elderly[Title/Abstract])) OR (("Middle Aged"[Mesh]) OR (Middle Age[Title/Abstract])))) AND (("Quality of Life"[Mesh]) OR ((((Life Quality[Title/Abstract]) OR (Health-Related Quality Of Life[Title/Abstract])) OR (Health Related Quality Of Life[Title/Abstract])) OR (HRQOL[Title/Abstract])))) AND (((("Exercise"[Mesh]) OR (((((((((((((((((((((((((Exercises[Title/Abstract]) OR (Physical Activity[Title/Abstract])) OR (Activities, Physical[Title/Abstract])) OR (Activity, Physical[Title/Abstract])) OR (Physical Activities[Title/Abstract])) OR (Exercise, Physical[Title/Abstract])) OR (Exercises, Physical[Title/Abstract])) OR (Physical Exercise[Title/Abstract])) OR (Physical Exercises[Title/Abstract])) OR (Acute Exercise[Title/Abstract])) OR (Acute Exercises[Title/Abstract])) OR (Exercise, Acute[Title/Abstract])) OR (Exercises, Acute[Title/Abstract])) OR (Exercise, Isometric[Title/Abstract])) OR (Exercises, Isometric[Title/Abstract])) OR (Isometric Exercises[Title/Abstract])) OR (Isometric Exercise[Title/Abstract])) OR (Exercise, Aerobic[Title/Abstract])) OR (Aerobic Exercise[Title/Abstract])) OR (Aerobic Exercises[Title/Abstract])) OR (Exercises, Aerobic[Title/Abstract])) OR (Exercise Training[Title/Abstract])) OR (Exercise Trainings[Title/Abstract])) OR (Training, Exercise[Title/Abstract])) OR (Trainings, Exercise[Title/Abstract]))) OR (("Resistance Training"[Mesh]) OR (((((((((((((((((((((((Training, Resistance[Title/Abstract]) OR (Strength Training[Title/Abstract])) OR (Training, Strength[Title/Abstract])) OR (Weight-Lifting Strengthening Program[Title/Abstract])) OR (Strengthening Program, Weight-Lifting[Title/Abstract])) OR (Strengthening Programs, Weight-Lifting[Title/Abstract])) OR (Weight Lifting Strengthening Program[Title/Abstract])) OR (Weight-Lifting Strengthening Programs[Title/Abstract])) OR (Weight-Lifting Exercise Program[Title/Abstract])) OR (Exercise Program, Weight-Lifting[Title/Abstract])) OR (Exercise Programs, Weight-Lifting[Title/Abstract])) OR (Weight Lifting Exercise Program[Title/Abstract])) OR (Weight-Lifting Exercise Programs[Title/Abstract])) OR (Weight-Bearing Strengthening Program[Title/Abstract])) OR (Strengthening Program, Weight-Bearing[Title/Abstract])) OR (Strengthening Programs, Weight-Bearing[Title/Abstract])) OR (Weight Bearing Strengthening Program[Title/Abstract])) OR (Weight-Bearing Strengthening Programs[Title/Abstract])) OR (Weight-Bearing Exercise Program[Title/Abstract])) OR (Exercise Program, Weight-Bearing[Title/Abstract])) OR (Exercise Programs, Weight-Bearing[Title/Abstract])) OR (Weight Bearing Exercise Program[Title/Abstract])) OR (Weight-Bearing Exercise Programs[Title/Abstract])))) OR (("Endurance Training"[Mesh]) OR (Training, Endurance[Title/Abstract])))) AND (((((Randomized controlled trial[Publication Type]) OR (randomized[Title/Abstract])) OR (trial[Title/Abstract])) OR (randomised[Title/Abstract])) OR (placebo[Title/Abstract]))
