# Supplementary material for: Effect of exercise intervention on health-related quality of life in middle-aged and older people with osteoporosis: a systematic review and meta-analysis
Source: PeerJ. 2025 Jan 31;13:e18889. doi: 10.7717/peerj.18889 (PMC11789655; doi:10.7717/peerj.18889)
Supplement: Supplemental Information 3 [file peerj-13-18889-s003.docx]

## Systematic Review and/or Meta-Analysis Rationale

For systematic reviews / meta-analyses, authors need to provide the following information:

1.The rationale for conducting the systematic review / meta-analysis.

**Reply:** In recent years, several studies have investigated the impact of exercise interventions on HRQOL among middle-aged and older patients with osteoporosis, but the conclusions have reached no agreement. The aim of this study was to integrate available evidence to identify the true significance of exercise interventions on HRQOL of middle-aged and older osteoporosis patients and to assess what type of exercise prescription was the best option.

2.The contribution that it makes to knowledge in light of previously published related reports, including other meta-analyses and systematic reviews.

**Reply:** The previous reviews were only qualitative systematic evaluations and no meta-analysis was performed. Thus, our study provides stronger evidence to summarize and confirm the true significance of exercise interventions on HRQOL in middle-aged and older patients with osteoporosis.
